# The two-year course of late-life depression; results from the Netherlands study of depression in older persons

**DOI:** 10.1186/s12888-015-0401-5

**Published:** 2015-02-12

**Authors:** Hannie C Comijs, Jasper Nieuwesteeg, Rob Kok, Harm W van Marwijk, Roos C van der Mast, Paul Naarding, Richard C Oude Voshaar, Peter Verhaak, Margot WM de Waal, Max L Stek

**Affiliations:** 1 Department Psychiatry/EMGO Institute for Health and Care Research VU University Medical Center/GGZinGeest, Amsterdam, The Netherlands; 2 GGZinGeest, Amsterdam, The Netherlands; 3 Parnassia/BAVO groep, Department of Old-age Psychiatry, The Hague, The Netherlands; 4 VU University Medical Center, Department of General Practice and Elderly Care Medicine/EMGO Institute for Health and Care Research, Amsterdam, The Netherlands; 5 Department of Psychiatry, Leiden University Medical Center, Leiden, The Netherlands; 6 GGNet, Department of Old-age Psychiatry, Apeldoorn/Zutphen, The Netherlands; 7 Department of Psychiatry, Radboud University Nijmegen Medical Center, Nijmegen, The Netherlands; 8 University of Groningen, University Medical Center Groningen, Interdisciplinary Center for Psychopathology of Emotion regulation (ICPE), Groningen, The Netherlands; 9 Department General Practice, University of Groningen, University Medical Center Groningen, Groningen, the Netherlands; 10 NIVEL, Netherlands Institute of Health Services Research, Utrecht, the Netherlands; 11 Department of Public Health and Primary Care, Leiden University Medical Center, Leiden, The Netherlands

**Keywords:** Late-life depression, Course, Determinants, Cohort study, Longitudinal

## Abstract

**Background:**

We aimed to examine the course of depression during 2-year follow-up in a group clinically depressed older persons. Subsequently, we studied which socio-demographic and clinical characteristics predict a depression diagnoses at 2-year follow-up.

**Methods:**

Data were used from the Netherlands Study of Depression in Older persons (NESDO; N = 510). Diagnoses of depression DSM-IV-TR criteria were available from 285 patients at baseline and at 2-year follow-up. Severity of the depressive symptoms, as assessed with the Inventory of Depressive Symptoms (IDS), was obtained from 6-monthly postal questionnaires. Information about socio-demographic and clinical variables was obtained from the baseline measurement.

**Result:**

From the 285 older persons who were clinically depressed at baseline almost half (48.4%) also suffered from a depressive disorder two years later. Patients with more severe depressive symptoms, comorbid dysthymia, younger age of onset and more chronic diseases were more likely to be depressed at 2-year follow-up. 61% of the persons that were depressed at baseline had a chronic course of depressive symptoms during these two years.

**Conclusions:**

Late-life depression often has a chronic course, even when treated conform current guidelines for older persons. Our results suggest that physical comorbidity may be candidate for adjusted and intensified treatment strategies of older depressed patients with chronic and complex pathology.

## Background

Late-life depression is a complex mood disorder with various etiological pathways [1] and high comorbidity with psychiatric and physical diseases, and cognitive decline [2-5]. Late-life depression often has a chronic course and high relapse rates [6-15], probably worse compared to younger age groups [16]. Previous studies were predominantly performed in community based or primary care samples, and some of them were targeting depressive symptoms or sub threshold depression, and not depression diagnoses according to formal diagnostic criteria. However, Beekman et al. [6] showed a gradient with respect to the prognosis of late-life depression, in which those with sub threshold disorders had the best outcome, followed by those with major depressive disorder (MDD), dysthymia and double depression (MDD and dysthymia). Only a few studies investigated the naturalistic course of late-life-depression in a large sample of older persons with formal depression diagnoses. Magnil et al. [15] observed the two-year course of depression in a cohort of primary care patients aged 60 years and older and found that, 15 of the 51 depressed patients (29%) had a remitting course, 25 (49%) remained depressive, and 11 (22%) had a fluctuating course. Hybels et al. [13] were the first to study the course of severe depression in older patients. They found that it took patients with a double depression longer to reach partial or full remission, and that they had higher MADRS (Montgomery–Åsberg Depression Rating Scale) scores after 3 years, compared to those with major depression alone. So, the results suggest that the course of late-life depression in patients from mental health institutions may be as poor as in patients from general practitioners or community based samples. However, more studies among clinically depressed patients are necessary to confirm this assumption.

For a better scientific and clinical understanding of the poor prognosis of late-life depression, it is important to study the clinical determinants of its course. This may help us to improve the treatment of late-life depression and to develop tailor made interventions. Among younger adults, clinical characteristics of the depression such as the severity of the depressive disorder, comorbid anxiety symptoms and age of onset are consistently found to be important predictors of the course [16-18]. Increased time to recovery from late-life depression is previously found to be associated with severity of depressive symptoms [19], but also with chronicity, later age of onset, cognitive decline [19,20] and medical comorbidity [21]. To date there are few longitudinal studies that included sufficient numbers of clinically depressed older persons enabling to study the course and determinants of the course of late-life depression. In the Netherlands Study on Depression in Older Persons (NESDO) depressed patients were included from both mental health care facilities and general practitioners, thus including depressed patients in various developmental and severity stages [22]. We now have 2-year follow-up data available, which offers us the possibility to study the two-year course of late-life depression and its determinants in our cohort.

The aims of the present study were twofold. First, we examined the course of depression during 2-year follow-up in a sample of clinically depressed patients, and second we studied which socio-demographic and clinical characteristics predicted a depression diagnoses at 2-year follow-up. Based on the literature we expected to find a high percentage of persons that are also depressed after 2-year, and that the severity of the depression and physical comorbidity would be important determinants of the poor outcome.

## Methods

### Participants

The Netherlands Study of Depression in Older persons (NESDO) is an ongoing multi-site cohort study designed to examine the (determinants of the) course and consequences of depressive disorders in older persons (≥60 years). Detailed description of the design and study sample is given in Comijs et al. [22]. In short, NESDO included 378 depressed patients (having MDD, dysthymia or minor depression according to DSM-IV criteria) and 132 non-depressed adults, aged 60 through 93 years. Participants were recruited in five regions in the Netherlands from both mental health care facilities and general practitioners. Participants were excluded when they had a dementia diagnosis or were suspected for dementia based on clinician’s judgement. In addition, to be sure that participants were able to fully understand and answer the questions, they were only included when they had a Mini Mental State Examination-score (MMSE) [23] of 18 or higher (out of 30 points), and when they had sufficient command of the Dutch language. The response rate of the depressed persons from the mental health institutions was estimated 48.7%, and from the general practices 60.3% [22]. Non-depressed comparisons were recruited from general practitioners (response rate 66.7%), and were included when they had no lifetime diagnosis of depression, dementia or other serious psychiatric disorders, and good command of the Dutch language [22]. The overall sample of 510 persons had a mean age of 70.6 years (SD: 7.3; range 60–93) and consisted of 331 (64.9%) women and 179 (35.1%) men. The mean level of education was 11.0 years (SD = 3.6; range 5–18 years). The majority of the sample had the Dutch nationality (99.4%). The depressed persons did not differ from the non-depressed comparison group with respect to mean age and sex, but they had a lower level of education, were more often divorced or widowed, and had a lower score on the MMSE [22].

### Materials and procedure

#### Data collection

Data collection of the baseline NESDO measurement started in 2007 and was finished in September 2010. It included an extensive assessment of psychopathology, socio-demographic characteristics, physical health and physical health markers, cognitive functioning, psycho-social functioning, and life style variables. The course of late-life depression was followed up every 6 months by means of a postal assessment, including questionnaires on the severity of depressive symptoms and physical health in the past 6 months, incident (chronic) stressors and functional limitations, and use of medications and health care. The questionnaires were the same questionnaires that were used during the face-to-face assessments [22]. A second face-to-face assessment was performed 2 years after the baseline assessment. It started in 2009 and was completed in September 2012. It consisted of all baseline measures (determinants and outcome variables) that were open to change, such as severity of psychopathology and diagnostics. Well-trained research assistants, mainly consisting of psychologists and mental health care nurses, conducted the interviews. All interviews were audio taped and were regularly controlled for their quality.

#### Ethical issues

The study protocol of NESDO has been approved centrally by the Ethical Review Board of the VU University Medical Center, and subsequently by the local ethical review boards of the Leiden University Medical Center, University Medical Center Groningen and the Radboud University Medical Center in Nijmegen. Written informed consent was obtained from all participants at the start of the baseline assessment. Written informed consent was asked for participating in the study, for permission to use genetic information, to retrieve medical information from the GP’s, and to link information to external databases. A privacy protocol has been developed in which confidentiality of data is guaranteed by using a unique research ID number for each respondent, which enables to identify individuals without using their names. Only the data manager has access to the record that links the ID number with the name of the participant [22]. All data are available on request (see http://nesdo.amstad.nl/).

#### Course of depression

Diagnoses of major depression, dysthymia and minor depression according to DSM-IV-TR criteria [24] at baseline and at two-year follow-up were assessed with the Composite International Diagnostic Interview (CIDI; WHO version 2.1). The CIDI is a structured clinical interview that is designed for use in research settings and has high validity for depressive and anxiety disorders [25,26]. Questions were added to determine the DSM-IV research diagnosis of current minor depression [22].

More detailed information about the severity of the depressive symptoms was obtained from the postal questionnaires, that were send to the respondents every 6 months. Severity of the depressive symptoms was assessed with the Inventory of Depressive Symptoms (IDS) [27]. The IDS is a 30-item self-report scale that was developed to carefully assess all core criterion diagnostic depressive symptoms. The scale has acceptable psychometric properties in depressed outpatients e.g. [27,28] and depressed inpatients [29]. The IDS is sensitive to both change over time and to differences between treatment conditions [30]. Chronbach’s alpha for the IDS in our sample was 0.83. The IDS was also included in the baseline and 2-year follow-up assessment, resulting in a total of 5 IDS ratings per participant. The IDS-scores range between 0 and 84, and is categorized according to severity as; < 14: no depression, 14–25: mild depression, 26 – 38 moderate depression, 39–48: severe depression and ≥ 49: very severe depression. Course types of depressive symptoms were computed from patients from whom we had at least 4 out of 5 IDS scores. We distinguished 5 course types:

1. remission, defined as at least the last two observations IDS score < 14,
2. intermittent depression, defined as at least one of the observations IDS < 14 (not being the last two observations),
3. chronic depression, defined as all IDS scores > 14 and 38 and sub classified as:
  - chronic mild to moderate depression, defined as all IDS scores between 14 and 26,
  - chronic moderate to severe depression, defined as all IDS scores between 26 and 84,
  - chronic depression with variable severity, defined as IDS scores varying between 14 to 84.

#### Determinants of depressive disorder at 2-year follow-up

Socio-demographic characteristics including age, sex, years of education, and partner status were assessed with standard questions. Sampling characteristics included sampling site (Amsterdam, Leiden, Groningen, Apeldoorn/Zutphen and Nijmegen) and sampling frame (primary care, ambulant health care and clinical health care).

Clinical variables included; first episode MDD (y/n), comorbid dysthymia (y/n), age of onset, comorbid anxiety disorder(s) (y/n), severity of depressive symptoms, cognitive functioning and number of chronic diseases. Information about the first episode MDD, recurrent MMD, dysthymia, and age of onset were all obtained from the CIDI (WHO version 2.1). Comorbid anxiety disorders (General Anxiety Disorder, Panic Disorder, Agoraphobia and Social Phobia) were also assessed using the CIDI. The Mini-Mental State Examination (MMSE) [23] was used to assess global cognitive functioning. The presence of chronic diseases was assessed by means of a self-report questionnaire. The participants were asked whether they currently or previously had any of the following chronic diseases or disease events: cardiac disease (including myocardial infarction), peripheral atherosclerosis, stroke, diabetes mellitus, COPD (asthma, chronic bronchitis or pulmonary emphysema), arthritis (rheumatoid arthritis or osteoarthritis), cancer, or any other chronic disease. The accuracy of self-reports of these diseases was compared to general practitioner information, and was shown to be adequate and independent of cognitive impairment [31]. Use of anti-depressive medication and benzodiazepines was determined by inspection of the medication that the participants brought in.

### Statistical analyses

Descriptive statistics were used to describe attrition and its determinants according to depression status at baseline. Next, diagnoses at 2-years follow-up were described according to baseline diagnostic status. In addition, specific course types were described according to the severity of depressive symptoms obtained from the five 6-monthly assessments with the IDS (see description IDS).

The socio-demographics, clinical and treatment characteristics were described for the depressed patients according to their depression diagnoses (MDD, dysthymia or minor depression) according to DSM-IV-R criteria at 2-year follow-up. Associations between baseline characteristics and the outcome measure depression diagnoses (y/n) at 2-year follow-up, were first assessed with univariate logistic regression analyses. Subsequently, when p < 0.10 the variables were entered in a final multivariate model. All analyses were performed by using SPSS 21.0 (IBM SPSS, Chicago, IL).

## Results

### Attrition and its determinants

From the 510 persons that were included at baseline, 401 persons participated in the 2-year follow-up assessment (overall attrition rate of 21.4%). Twenty-eight persons died during the two-year follow-up (5.5%). From the 482 participants who were still available for the study at that time point, 401 persons (83.4%) participated in second face-to-face measurement. In the patient group, the most important reasons for attrition were death (28.0%) and mental problems (37.6%). In the non-depressed comparison group the most important reason for attrition was, having no interest or no time (50%) (Table 1).

**Table 1:** **Attrition at 2-year follow-up according to depression status at baseline (n = 510)**

	Patient group (n = 378)	Control group (n = 132)
	N (%)	N (%)
Respondents at 2-y follow-up	285 (75.4)	116 (87.9)
Non-respondents at 2-y follow-up	93 (24.6)	16 (12.1)
*Reasons of attrition*		
Deceased	26 (28.0)	2 (12.5)
Refusal		
No interest/no time	14 (15.0)	8 (50.0)
Bad experience with previous interview	1 (1.1)	0 (0)
Unable		
Due to physical reasons	12 (12.9)	2 (12.5)
Due to mental reasons	35 (37.6)	4 (25.0)
Noncontact		
No contact	4 (4.3)	0 (0)
Moved abroad	1 (1.1)	0 (0)

Attrition was significantly higher among persons who were depressed at baseline, and among those with lower education, more severe psychopathology and lower cognitive functioning (all p < 0.05). Recruitment area and sampling frame also differed between respondents and non-respondents at follow-up. Non-respondents had more often been recruited in Apeldoorn/Zutphen and Nijmegen and from outpatient and inpatient mental health facilities (both p < 0.01).

### Course of depression

Depression diagnoses at two-year follow-up according to baseline depression diagnoses are shown in Table 2. From the 285 persons who were suffering from a depressive disorder at baseline, almost half (48.4%) also suffered from a depressive disorder two years later. About 59% of the persons with a double depression (MDD and dysthymia) at baseline, also had a depression diagnoses at 2-year follow-up. From the persons with a MDD at baseline 44% were also depressed at follow-up. All four persons with dysthymia only at baseline were also depressed at FU. Among the persons with a minor depression the highest remission rates were reached (63.6%).

**Table 2:** **Depression diagnoses at 2-year follow-up according to baseline diagnoses**

	N	2 year follow-up				
Baseline		Double depression ^ 1 ^	Major depression	Dysthymia	Minor depression	No depression diagnoses
Double depression^1^, n (%)	71	20 (28.2)	17 (23.9)	3 (4.2)	2 (2.8)	29 (40.8)
Major depression, n (%)	199	38 (19.1)	36 (18.1)	6 (3.0)	8 (4.0)	111 (55.8)
Dysthymia, n (%)	4	0	1 (25)	3 (75.0)	0	0
Minor depression, n (%)	11	0	1 (9.1)	2 (18.2)	1 (9.1)	7 (63.6)

^1^Major depression and dysthymia.

Only 19% of the persons that was depressed at baseline was completely in remission, with at least the last two IDS assessments lower than 14, whereas 56% of the persons with a depressive disorder at baseline, but without a depressive disorder at follow-up, still had IDS-score higher than 14, suggesting residual depressive symptoms at follow-up.

**Figure 1:**
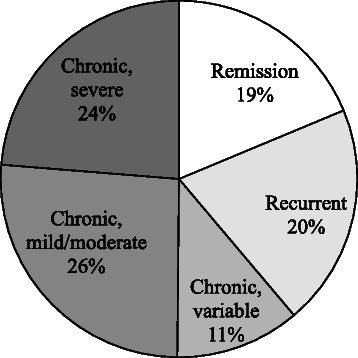
**Course of depression (percentages).** [Remission: at least the last two observations IDS score < 14; Intermittent: at least one of the observations IDS < 14 (not being the last two observations); Chronic depression, defined as all IDS scores > 14 and sub classified as: chronic mild to moderate depression, defined as all IDS scores between 14 and 26; chronic moderate to severe depression, defined as all IDS scores between 26 and 84; chronic depression with variable severity, defined as IDS scores varying between 14 to 84].

**Figure 2:**
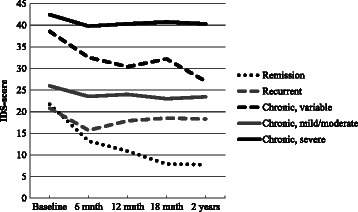
**Severity of depressive symptoms according to course during 2-year follow-up.** [Remission: at least the last two observations IDS score < 14; Intermittent: at least one of the observations IDS < 14 (not being the last two observations); Chronic depression, defined as all IDS scores > 14 and sub classified as: chronic mild to moderate depression, defined as all IDS scores between 14 and 26; chronic moderate to severe depression, defined as all IDS scores between 26 and 84; chronic depression with variable severity, defined as IDS scores varying between 14 to 84].

According to the severity of depressive symptoms as assessed with the IDS every six months, 61% of the persons that were depressed at baseline had a chronic (mild/moderate, severe, or variable) course (see Figures 1 and 2), whereas 20% had intermittent depression – with at least one assessment during the 2-year period without depressive symptoms (IDS score <14).

### Determinants of depressive disorder at 2-year follow-up

Finally, we examined which baseline socio-demographic and clinical characteristics predicted a depression diagnoses at 2-year follow-up (Table 3). Univariate analyses showed that dysthymia, a younger age of onset, higher IDS score, more chronic diseases and being recruited from primary care were associated with having a depressive disorder at follow-up. In multivariate regression analyses, independent associations appeared to be a younger age of onset, higher IDS score, and having more chronic diseases at baseline (Table 4).

**Table 3:** **Descriptives of patient who were depressed at baseline according to their depression status at 2-year follow-up**

	Not depressed at follow-up (n = 147)	Depressed at follow-up (n = 138)
*Socio-demographics at baseline*		
- Mean age (sd)	70.4 (7.1)	70.9 (7.9)
- Female gender, n (%)	97 (66.0)	90 (65.2)
- Years of education, mean (sd)	10.7 (3.2)	10.5 (3.7)
- No partner, n (%)	66 (44.9)	72 (52.2)
- Sampling site, n (%)		
- Amsterdam	61 (48.8)	64 (51.2)
- Leiden	26 (44.1)	33 (55.9)
- Groningen	22 (55.0)	18 (45.0)
- Apeldoorn/Zutphen	21 (67.7)	10 (32.2)
- Nijmegen	17 (56.7)	13 (43.3)
*Clinical characteristics at baseline*		
- First episode MDD, n (%)	70 (47.6)	65 (47.1)
- Dysthymia, n (%)	29 (19.7)	46 (33.3)
- Age of onset of depression, mean (SD)	51.2 (19.5)	44.2 (20.7)
- Comorbid anxiety disorder, n (%)	48 (32.7)	57 (41.3)
- Severity depression symptoms	25.6 (11.8)	33.9 (12.5)
Sampling frame, n (%)		
- Primary care	17 (40.5)	25 (59.5)
- Ambulant mental health care	111 (51.9)	103 (48.1)
- Clinical mental health care	19 (65.5)	10 (34.5)
- Use anti depressive medication, n (%)	106 (72.1)	96 (69.6)
- Use of benzodiazepines, n (%)	54 (36.7)	57 (41.3)
*Comorbidity*		
- Number of chronic diseases, mean (sd)	1.8 (1.2)	2.4 (1.7)
- MMSE, mean (sd)	28.0 (1.7)	27.7 (1.8)

MMSE: Mini Mental State Examination.

**Table 4:** **Univariate and multivariate determinants of a depressive disorder (yes/no) at follow-up in the patient group (n = 285)**

	The presence of depression at 2-year follow up	
	Univariate	Multivariate ^ 1 ^
	OR (95% CI)	OR (95% CI)
*Socio-demographics*		
- Age at baseline, in years	1.01 (0.98 – 1.04)	
- Female gender	0.97 (0.59 – 1.58)	
- Education, in years	0.99 (0.92 – 1.05)	
- No partner	1.34 (0.84 – 2.13)	
- Sampling site		
- Amsterdam	Ref group	Ref group
- Leiden	1.21 (0.65 – 2.25)	1.63 (0.81-3.29)
- Groningen	0.78 (0.38 – 1.59)	0.95 (0.42-2.11)
- Apeldoorn/Zutphen	0.45 (0.20 – 1.04)	0.56 (0.21-1.53)
- Nijmegen	0.73 (0.33 – 1.63)	0.81 (0.32-2.06)
*Clinical characteristics at baseline*		
- First episode MDD	0.98 (0.62 – 1.56)	
- Dysthymia	**2.03 (1.19 – 3.49)**	1.30 (0.71-2.37)
- Onset of depression, in years	**0.98 (0.97 – 0.995)**	**0.99 (0.98-1.00)**
- Comorbid anxiety disorder	1.45 (0.90 – 2.35)	
- Severity depression symptoms	**1.06 (1.04 – 1.08)**	**1.05 (1.03-1.07)**
Sampling frame		
- Primary care	Ref group	Ref group
- Ambulant mental health care	0.63 (0.32 – 1.24)	0.57 (0.26-1.21)
- Clinical mental health care	**0.36 (0.13 – 0.96)**	0.43 (0.13-1.42)
- Use anti depressive medication	0.88 (0.53 – 1.47)	
- Use of benzodiazepines	1.05 (0.83 – 1.34)	
*Comorbidity at baseline*		
- Number of chronic diseases	**1.37 (1.16 – 1.63)**	**1.21 (1.01-1.46)**
- MMSE	0.91 (0.80 – 1.04)	

^1^All variables with univariate p < 0.10 included.

MMSE: Mini Mental State Examination.

P-levels < 0.05 are printed bold.

## Discussion

Our study showed that in a sample of clinically depressed older patients nearly 50% still had a depression diagnoses at 2-year follow-up. Of our patients 61% showed a chronic course of the depressive symptoms during the two-year period. Patients with more severe depressive symptoms, comorbid dysthymia, younger age of onset and more chronic diseases were more likely to be depressed at 2-year follow-up.

Our findings are largely in line with expectations from community based, primary care and other clinical samples of older persons [6,10,13,15,32]. Consistent with the findings of Hybels et al. [13], we found that the persons with a double depression (MDD and dysthymia) had the poorest prognosis, with 59% still suffering from a depressive disorder at two years follow-up. Compared to studies among adults aged 18 to 65 years, our remission rates seem somewhat lower. In the Netherlands Study on Depression and Anxiety (NESDA) [18], which has a comparable design and uses largely the same instruments as in NESDO, about 80% of the purely depressed patient reached remission within 2 years, whereas from the persons with a comorbid anxiety disorder only 50% reached remission within that time frame. In our study, 36.8% of the depressed persons had a comorbid anxiety disorder, however, comorbidity was no predictor of a depression diagnoses at follow-up. Thus, we may conclude that our study confirms the poorer prognosis of depression in terms of chronicity among older persons compared to younger adults.

Since we assessed the severity of depressive symptoms every 6 months, it was possible to study the course of depression in more detail. Of the depressed patients, 61% showed a chronic course of the depressive symptoms during the two years of follow-up, whereas 20% had intermittent depressive symptoms. These findings suggest that most patients had clinically relevant levels of depressive symptoms all the time during this 2-year period, further stressing the persistence and chronicity of the depressive symptoms, despite the fact that most of them were being treated in mental health care facilities. Only 19% of the depressed older people reached complete remission, whereas 56% of the persons without a depression diagnoses at follow-up still had residual depressive symptoms.

With respect to the determinants of the prognosis of depression we found that patients with more severe depression at baseline, comorbid dysthymia, younger age of onset and more chronic diseases were more likely to be depressed at 2-year follow-up. None of the socio-demographic variables appeared to be a predictor of the prognosis, neither was comorbid anxiety disorder or cognitive functioning. Our findings are partly in line with previous studies that reported severity and chronicity of depressive symptoms [19] and medical comorbidity [21] to be related with an increased time to recovery. In contrast with Alexopoulos [19] however, we found an early onset of depression to be associated with poor prognosis. Also in contrast with our results, Bogner [14] showed in the PROSPECT study that married patients had a favourable course of depression, suggesting that depressed persons with a supportive relationship improve more quickly. In our study, partner status was not statistically significant. This may be the due to the severity of depression, our sample was mainly recruited in in- and outpatients facilities, whereas the PROSPECT sample was recruited in primary care.

Although we included important socio-demographic, and clinical characteristics as possible determinants for the prognosis of depression, additional key biological, health and psychosocial determinants may be of relevance for the prognosis of depression. However, before conducting such in-depth analyses in the NESDO sample, we needed detailed insight in the course of late-life depression and its socio-demographic and clinical determinants, as was the aim of the present paper.

Attrition is an inevitable problem in studies among vulnerable older persons. We made extensive efforts to contact and invite persons to participate in the study and offering them shortened interviews when necessary. We kept in touch with all participants every half year and send them yearly newsletters. Nevertheless, the attrition at 2-year follow-up was highest in the depressed group 24.6% compared to 12.1% in the non-depressed control group. In the depressed group 28% died and 37.6% did not want to participate due to mental reasons. Unfortunately attrition was selective; attrition was higher among persons who were depressed at baseline and who had severe psychopathology, lower cognitive functioning, and were recruited from outpatient or inpatients mental health care settings. In the aforementioned comparable NESDA study among younger adults aged 18–65, the two-year attrition rate was 12.9% which was relatively low compared to other epidemiological studies in psychiatric samples and was mainly due to refusal to further participate [33]. Among older adults, attrition rates are expected to be higher, because of a higher risk for death and diseases compared to younger adults. In the Longitudinal Aging Study Amsterdam, a population based cohort study among older persons age 55 years and older, three-year attrition rates were around 19% and was mainly due to death [34]. We may therefore conclude that the attrition rate in our study is not extremely high, when taking age and disease status of our sample into account, but it may limit the generalizability of the findings to some extent and needs to be reflected upon in future studies.

It should be noted that our findings cannot be generalized to community-dwelling older persons, as most of our patients were recruited from specialized mental health facilities and may represent a group with more refractory depression at baseline. However, we were especially interested in this group because patients with clinical depression are often underrepresented in community based samples. Thus far, few studies investigated the naturalistic course of late-life-depression in a large sample of older persons with formal depression diagnoses. Our findings are therefore important for clinical practice.

### Clinical implications

As most of the diagnosed patients (85.3%) were under treatment when they entered the NESDO study, the results may tell us something about the adequacy of the depression treatment in this older age group. Regular interventions are mainly adapted from guidelines that are based on research performed in younger adults, assuming that depression in older persons has the same underlying mechanism as depression in younger adults. Although pharmacological and psychotherapy are effective treatments for late-life depression [35,36], it is suggested that antidepressants may be less efficacious in in older depressed patients compared to younger ones [37,38]. Moreover, studies are generally limited to the youngest old, reflected by average samples ages below 70 years and minimal physical comorbidity [36].

In older persons, depression treatment may need to be tailored to address underlying etiological factors and comorbidity as well. The group of Alexopoulos [39] developed a personalized intervention for depressed patients with severe chronic obstructive pulmonary dysplasia (COPD) and showed that this intervention reduced depressive symptoms and dyspnea-related disability more than usual care over 28 weeks. However, thus far there is only limited evidence that such a multifactorial personalized treatment is more effective than the regular treatment. Nevertheless, personalizing depression treatment seems necessary to improve the treatment of depression, especially in this older age group. Our results suggest that physical comorbidity may be candidate for adjusted and intensified treatment strategies of older depressed patients with chronic and complex pathology.

## Conclusions

Our study showed that almost half of a group of older patients with a depressive disorder were also suffering from a depressive disorder two years later, and that most of them had a chronic course of the depressive symptoms during the 2 years of follow-up. More serious depression, a younger age of depression onset, and more somatic comorbidity were independent determinants of a poor prognosis of depression.
